# The Relationship between Canopy Cover and Colony Size of the Wood Ant *Formica lugubris* - Implications for the Thermal Effects on a Keystone Ant Species

**DOI:** 10.1371/journal.pone.0116113

**Published:** 2014-12-31

**Authors:** Yi-Huei Chen, Elva J. H. Robinson

**Affiliations:** York Centre for Complex Systems Analysis, Department of Biology, University of York, Wentworth Way, York, YO10 5DD, United Kingdom; University of Sussex, United Kingdom

## Abstract

Climate change may affect ecosystems and biodiversity through the impacts of rising temperature on species’ body size. In terms of physiology and genetics, the colony is the unit of selection for ants so colony size can be considered the body size of a colony. For polydomous ant species, a colony is spread across several nests. This study aims to clarify how climate change may influence an ecologically significant ant species group by investigating thermal effects on wood ant colony size. The strong link between canopy cover and the local temperatures of wood ant’s nesting location provides a feasible approach for our study. Our results showed that nests were larger in shadier areas where the thermal environment was colder and more stable compared to open areas. Colonies (sum of nests in a polydomous colony) also tended to be larger in shadier areas than in open areas. In addition to temperature, our results supported that food resource availability may be an additional factor mediating the relationship between canopy cover and nest size. The effects of canopy cover on total colony size may act at the nest level because of the positive relationship between total colony size and mean nest size, rather than at the colony level due to lack of link between canopy cover and number of nests per colony. Causal relationships between the environment and the life-history characteristics may suggest possible future impacts of climate change on these species.

## Introduction

Climate change is one of the most notable ecological and environmental issues. This phenomenon, which is of global concern, has altered species distribution and abundance, and consequently affected ecosystems and biodiversity [1]–[4]. There are many predictions for climate change, including more frequent storms and hurricanes, and greater snowfall. Rising average and extreme temperatures are the main and general predictions [1]. For plants and many ectotherms, temperature has a profound impact on many functions relating to an organism’s size, such as metabolic rates and rates of gas exchange [5]. Body size is probably the most significant life-history characteristic of an animal due to its influence on most physiological and morphological characters [5]–[7]. Therefore, climate change may affect animals through impact on body size mediated by rising temperature [8], [9].

For social insects, the colony can be considered the biological analogue of the body of a solitary organism [10]–[12]. Colony size of social insects has been represented by the total number of individuals or workers in a colony [10]–[12]. Just as body size has a significant role for solitary organisms, colony size has been known to correlate with the lifestyle of a social insect colony, for example, competitive abilities, foraging behaviours and life span [13]–[17]. Again, just as for body size, temperature is one of the exogenous factors which affects colony size in social insects [18], [19]. For these reasons, colony size could be a useful index to understand how climate change will influence social insects.

The red wood ants are a group of morphologically similar *Formica* species [20], [21], which are ecologically dominant and have impacts at multiple community levels including ants, other arthropods and vertebrates, across northern Eurasia [22]–[28]. Red wood ants can affect the growth of trees both negatively, by herding sap-sucking aphids, and positively, by increasing predation or harassment of other herbivores [29], [30]. They build nests with large aboveground mounds which function as habitats for myrmecophiles and influence the nutrient cycle of the forest [31]–[35]. They are also ecological indicators for land-use changes in European broadleaf forest and taiga [36]. Red wood ants have significant impacts on forest ecosystems and most of them are considered “near threatened” by the International Union for Conservation of Nature [37]. Furthermore, because future climate change predictions also indicate more severe warming at higher latitudes [38], [39], understanding how climate change may affect these temperate species is therefore important for future conservation actions.

Species distribution modelling and physiological experiments have been the prevailing research for the potential effects caused by climate change. Temperature experiments such as testing thermal tolerance can be an useful tool for modelling and predicting responses of ants to warming [40]. For ants, some species-level studies have asserted the negative impacts on physiology or behaviours from climate change [40]–[42]; others have revealed its promotive role on the expansion of species distribution, especially for invasive species [43]–[48].

Although species’ responses to specific environmental factors such as temperature can be tested in laboratories, a laboratory approach may not be effective for capturing the effects caused by daily or annual dynamics of temperature. It would be more comprehensive if we can directly investigate these in the field, if conditions accurately representing the natural environment cannot be simulated. This could be achieved by a field transplant or a common garden experiment [49]. However, as for many social insects, red wood ant nests are complex and long-lasting. Wood ants spend many years building large nest mounds in woodland, and one red wood ant colony may also settle in several spatially separated but socially connected nests, called polydomy [50], [51]. It is not feasible to move the whole colony without damage and long-term effects on the colony’s function and organisation.

Fortunately, it is known that the thermal environments of the locations on a woodland floor are strongly influenced by canopy cover [52], [53]. This provides a practicable approach to explore how colony size and nest size are related to a lasting but localised thermal environment, which a red wood ant colony may continually experience for years. Moreover, in addition to temperature, higher canopy cover may imply more surrounding trees, which probably provide more aphids, the main food resource of red wood ants. Food resource availability may positively relate to wood ant nest size [54], [55]. Therefore, we might be able to detect the role of food resource availability in the relationship between canopy cover and nest size.

In this study, we investigated the relationship between canopy cover and both the total colony size (worker population of a polydomous colony) and nest size (worker population of a single nest) of a woodland specialist ant species in the field. There is a known negative relationship between canopy cover and temperature [52], [56], [57]; we verified this at our site by collecting thermal data at the colony locations. Larger nests or colonies are expected to cope better with colder environments due to increased abilities to regulate inner nest temperature [54], [58]. We would therefore expect to observe larger colony size and nest size in shady areas with a colder environment.

## Materials and Methods

### Species and location

The study species was the red wood ant *Formica lugubris* (Hymenoptera: Formicidae). To focus on the relationship between canopy cover and colony size, and to minimise the effects from altitude and slope direction, we conducted our study in a part of the Longshaw Estate, Peak District (53°18′35″N, 01°36′25″W; access permission obtained with S. Ellis by the National Trust) in the UK. It is a flat area (∼1.1 km^2^) with an altitudinal range of 270–350 m. *Formica lugubris* has both monodomous and polydomous social forms [50], [59], [60], and is polydomous in Great Britain [50], [61]. We defined a polydomous colony as a group of nests which are connected each other by trails. There are over 900 nests of polydomous *F. lugubris* in our sampling area, and the number of nests per colony ranging from 1 to 22 nests [62].

### Methods

The study was conducted in June 2013, when canopy cover had reached a relatively stable level. To choose colonies to include in our sample, we divided the experimental site into a grid of 44 squares with a side length each of 140 metres. We defined the intersections of the gridlines as our sampling points. We located the nest nearest to each sampling point and the colony to which this nest belonged was chosen for inclusion. Because the longest distance between two nests of the same polydomous colony was 52 metres (2.5 metres on average, more than 90% trails below 8 metres, S. Ellis, preliminary survey), by this method, we minimised the chance of choosing a colony that included several nests within different sampling points. We defined a sampling point as having no colony present if we could not find any nest within a radius of 70 metres from the intersection. This sampling method was able to include a range of canopy cover (from an isolated tree to dense cover).

We mapped the chosen colonies, recording: the number of and size of nests; spatial distribution pattern of nests; the trails between nests; foraging trails between nests and trees. In addition, number of inter-nest trails per nest, trail length and number of forage trees used by each nest were recorded. In our study, we defined a distinct trail from a nest to a tree as a foraging trail (see [62]). However, it does not mean that the nests without any obvious foraging trails were not foraging at all; they might be involved in other foraging activities. A Mound-Volume method was used to estimate nest size; three dimensions of nest mound were multiplied to represent the total number of individuals of mound-building wood ants [54], [63], [64]. This method has been tested and shown to provide a reliable estimate of nest worker population in this species [63]. A photo was taken skyward above each nest using 180-degree hemispherical lens (FC-E8 fisheye lens with Coolpix 5000, Nikon Corporation, Tokyo, Japan) which produces circular images that record the size, shape, and location of gaps of the canopy. Canopy cover (percentage) was estimated from the circular photo using the software Gap Light Analyzer 2.0 [65].

For the background thermal environment, we derived annual solar radiation data from digital elevation model data at 10-metre resolution (Crown Copyright 2014. An Ordnance Survey/EDINA supplied service.). The calculation was done using the Area Solar Radiation tool in the Spatial Analyst toolbox of ArcMap 10.1 and specifying the latitude, elevation and slope direction of our sampling points. The calculation sampled every day throughout 2013, using a 30-minute interval. All other settings were set to default. Besides the annual solar radiation as background data, we also wanted to obtain information about the small-scale thermal environment of the nest. For this reason, a temperature-recording device was placed on the ground next to the north side (to reduce the chance of direct sunshine exposure) of the nest which was discovered first in every colony. The devices consisted of a polyethylene terephthalate (PET) tube (diameter = 10 cm, length = 20 cm) wrapped in aluminium foil to reduce the effect of direct solar radiation [66]. A thermal datalogger (iButton: DS1921G-F5; Maxim/Dallas Semiconductor, TX, US) was placed in each device to record hourly environmental temperatures from 31^st^ May for 16 days.

### Statistical analyses

Total colony size was calculated as the sum of nest sizes to represent the total number of individuals in a colony. Size data (nest size and total colony size) were transformed by log_10_ to normalize the distributions. We used “lme” function from the “nlme” package for R (version 3.0.1, R Development Core Team) to fit linear mixed-effect models for: 1) the effects of annual solar radiation and canopy cover on nest size; 2) the relationships of the number of nests per colony to canopy cover and nest size; 3) whether the presence or absence of foraging trail was related to canopy cover and nest size; and 4) the relationships of foraging trail length to canopy cover and nest size. For linear mixed-effect models, the best model was selected according to AIC and the significance of factors. Colony identity was included as a random effect in the models.

Linear regression models were used for: 1) the effects of annual solar radiation and canopy cover on total colony size; 2) the relationship between the size of the largest nest of each colony and canopy cover, and between the size of the smallest nest of each colony and canopy cover; 3) the relationship between total colony size and mean nest size per colony; 4) the relationship of annual solar radiation, number of nests per colony and canopy cover to six local temperature parameters- the mean and the standard deviation of hourly temperature (Temp_Mean_ and Temp_SD_), the mean and the standard deviation of daily maximum and minimum temperature (Max_Mean_, Max_SD_, min_Mean_, and min_SD_). For linear regressions, F test was used to select the best model. Pearson’s correlation was used for total colony size and six local temperature parameters. If a temperature parameter was correlated to both total colony size and canopy cover, partial correlation was used to measure the degree of association between total colony size and canopy cover, with the effects of this temperature parameter removed.

To analyse the variation in nest size at different levels of canopy cover, nests were separated into three groups based on the canopy cover of their location to balance the sample size of each group: nests with canopy cover lower than 51.2% (n = 67), between 51.2% and 67.5% (n = 67), and higher than 67.5% (n = 67). To analyse the differences of total colony size between colonies, we also separated colonies into three groups based on their number of nests to balance the sample size: colonies with one to three nests (Close-to-Monodomous Group, n = 12), colonies with four to seven nests (Intermediate-Polydomous Group, n = 12), and colonies with more than seven nests (Polydomous Group, n = 10). Levene’s tests were used to compare the variances between groups. Kruskal-Wallis’ test was used to compare total colony size of each group. Linear regression model, Levene’s test and Kruskal-Wallis’ test were conducted with the JMP statistics package (version 6.0.0; SAS institute, Cary, NC, USA).

## Results

**Figure 1:**
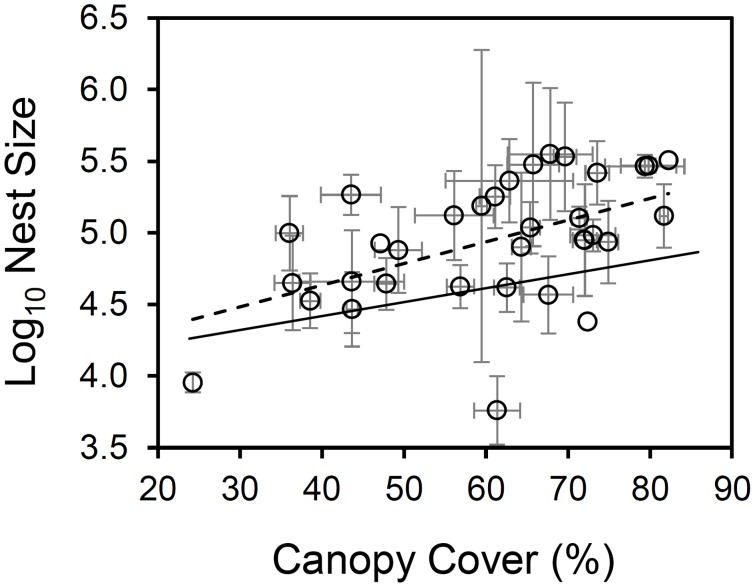
The relationship between mean nest size and mean canopy cover. Circle dots: the log10 mean nest size and mean canopy cover of 34 colonies; grey error bar: 1 SE, four points without error bars are colonies containing only one nest; dashed line: *y* = 0.0149*x*+4.0423, *F* ratio = 11.10, *P*<0.001, *r^2^* = 0.26, model fitted by linear regression for the relationship between mean nest size and mean canopy cover; solid line: *y* = 0.0097*x*+4.0282, from the fixed effects of the linear mixed-effect model, which includes colony identity as a random effect. Full analyses are showed in results.

**Figure 2:**
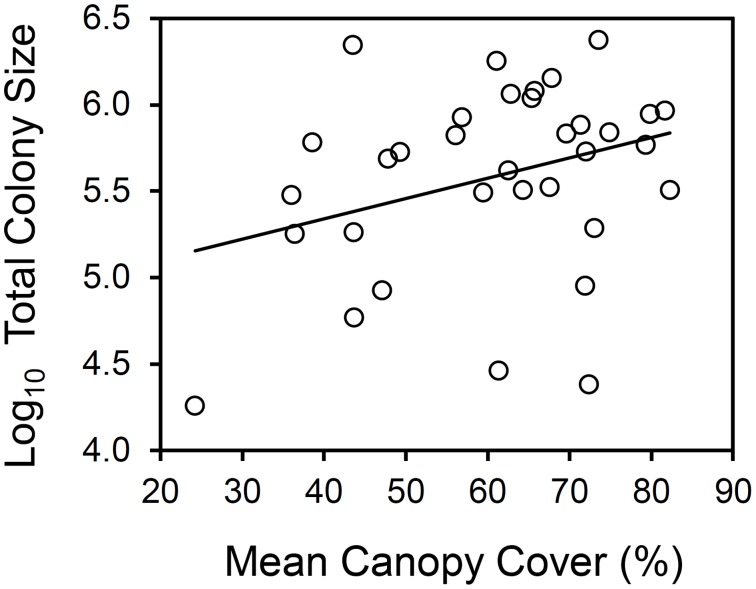
The trend between total colony size and mean canopy cover of 34 colonies. Linear regression, solid line: *y* = 0.0117*x*+4.8727, *F* ratio = 3.67, *P* = 0.06, *r^2^* = 0.10.

Thirty-four colonies, with a total of 201 nests, were sampled and recorded for this study. There was no colony at 10 sampling points. We found that nest size increased significantly with increasing canopy cover (linear mixed-effect model, solid line in Fig. 1, fixed effect: t = 2.19, *P*<0.05, n = 201, reduced model AIC = 464.23). The full model contained two factors: canopy cover and annual solar radiation, the latter factor had no significant effect on nest size (t = –0.69, *P* = 0.50, n = 201, full model AIC = 489.40). There was no significant relationship between the size of the largest nest of each colony and canopy cover (linear regression: *F* = 2.64, d.f. = 33, *P* = 0.11, see Fig. 1), and between the size of the smallest nest of each colony and canopy cover (linear regression: *F* = 3.14, d.f. = 33, *P* = 0.09, see Fig. 1). The variances of nest size did not significantly differ between three groups with different canopy cover (Levene’s test, *F* = 0.72, *P* = 0.49, n = 67 for each group). Total colony size, which was the sum of the size of all nests in that colony, borderline significantly increased with increasing mean canopy cover (linear regression: *F* = 3.67, d.f. = 33, *P* = 0.06, reduced model *r* ^2^ = 0.10, Fig. 2). Again, the factor annual solar radiation did not have a significant effect on colony size (t = –1.18, *P* = 0.25), and did not significantly improve the model (full model *r* ^2^ = 0.19, F test, *F* = 1.17, *P* = 0.25). Canopy cover at our 201 sampled nests ranged from 24% to 86%, with a mean of 59%.

**Figure 3:**
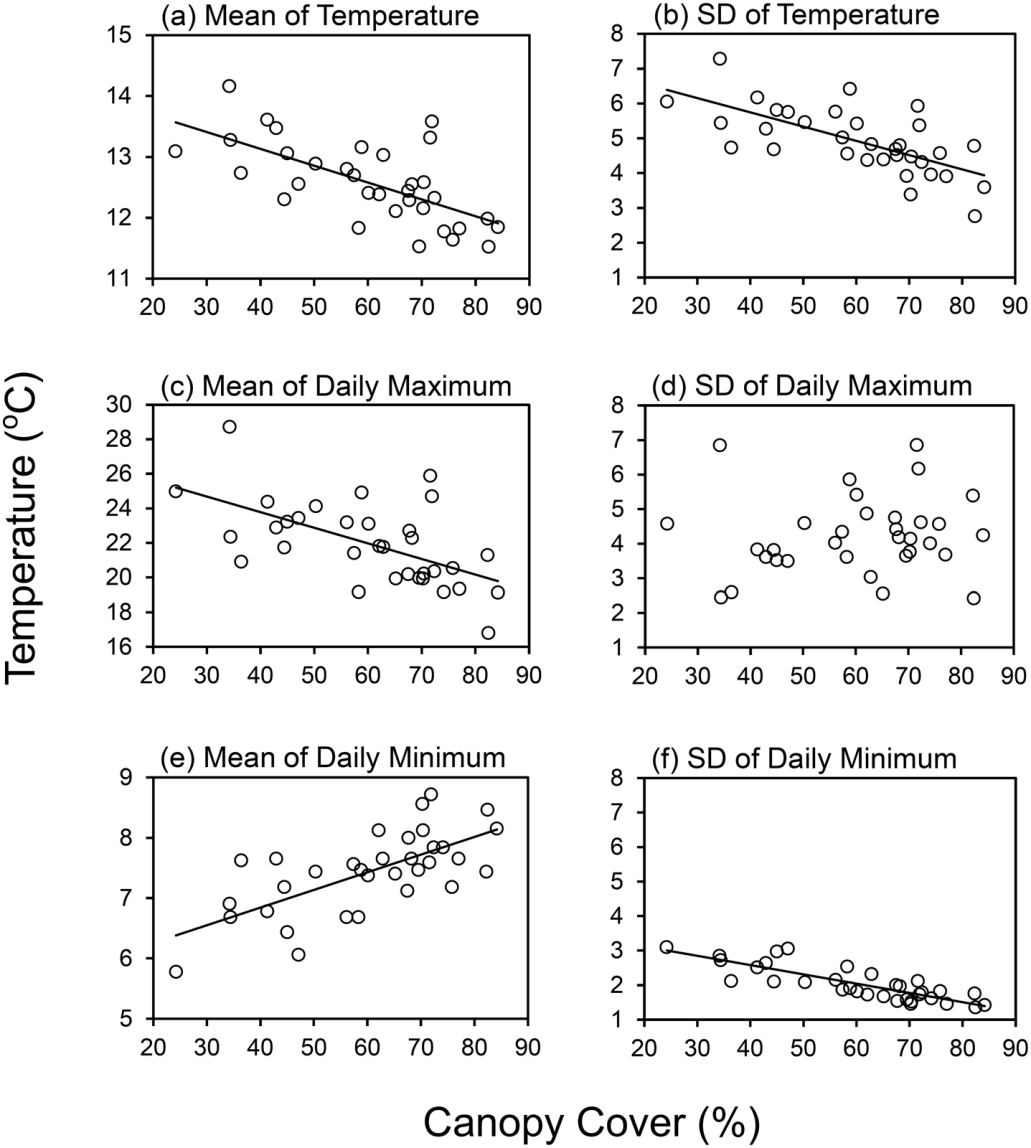
The relationships between canopy cover and six local temperature parameters for 33 colonies. Solid line of each graph shows the significant model fitted by linear regression. (a) the mean of temperature: *y* = –0.03*x*+14.24, *P*<0.001, *r^2^* = 0.43. (b) the standard deviation (SD) of temperature: *y* = –0.04*x*+7.38, *P*<0.001, *r^2^* = 0.46. (c) the mean of daily maximum temperature: *y* = –0.09*x*+27.39, *P*<0.001, *r^2^* = 0.34. (d) the SD of daily maximum temperature: not significant. (e) the mean of daily minimum temperature: *y* = 0.03*x*+5.67, *P*<0.001, *r^2^* = 0.46. (f) the SD of daily minimum temperature: *y* = –0.03*x*+3.66, *P*<0.001, *r^2^* = 0.69.

One temperature-recording device was lost. According to the records of the 33 nests from which dataloggers were retrieved, both Temp_Mean_ and the Temp_SD_ of local environmental temperatures were lower with increasing canopy cover (linear regressions: Fig. 3). Max_Mean_ were also lower in shadier areas, whereas there was no significant relationship between the Max_SD_ and canopy cover. Min_Mean_ increased with rising canopy cover, whereas min_SD_ decreased (Fig. 3). The relationships between the local temperature parameters and total colony size were similar to the relationships between the local temperature parameters and canopy cover: there were negative correlations of total colony size with Temp_Mean_, Temp_SD_, Max_Mean_ and min_SD_, whereas min_Mean_ was borderline significantly positively correlated with total colony size. There was no significant correlation between total colony size and Max_SD_ (Table 1). For three-way correlation between total colony size, canopy cover and the local temperature parameters, using partial correlation to remove the effects of the local temperature parameters eliminated the positive trend between total colony size and canopy cover (Table 1). Annual solar radiation levels had no significant relationship with the six local temperature parameters (Annual solar radiation: 873978.25±26008.40, whr/m^2^, Mean ± SD, linear regressions: *F* = 0.02–0.94, d.f. = 32, *P* = 0.33–0.89).

**Table 1:** Correlations and partial correlations between canopy cover, total colony size and six local temperature parameters (the mean and the standard deviation of hourly temperature, Temp_Mean_ and Temp_SD_; the mean and the standard deviation of daily maximum and minimum temperature, Max_Mean_, Max_SD_, min_Mean_, and min_SD_).

	Canopy Cover	Temperature Parameters		Partial Correlation^#^
Total Colony Size	0.31!	Temp_Mean_	–0.44*	0.10
		Temp_SD_	–0.40*	0.10
		Max_Mean_	–0.39*	0.16
		Max_SD_	–0.10	-
		min_Mean_	0.31!	0.14
		min_SD_	–0.45**	–0.13

! *P* = 0.08, *P<0.05, **P<0.01, ^#^Partial correlation between total colony size and canopy cover with the effects of local temperature parameters removed, n = 33.

**Figure 4:**
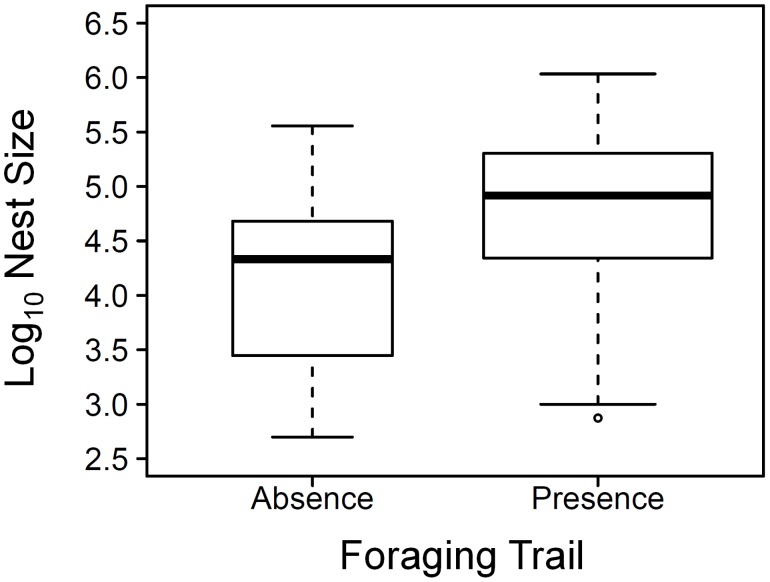
The relationship between nest size and the presence or absence of foraging trail. Boxplots show the range, quartiles, medium and outliers of the data. Boxplot width is proportional to the square root of sample size. This figure does not take colony identity in account, but the full analysis does (linear mix-effect model).

The sizes of nests with at least one foraging trail was greater than those of nests without any foraging trail (linear mixed-effect model, fixed effect: t = 4.70, *P*<0.001, n = 201, model AIC = 441.93, Fig. 4). Nests with foraging trail/s were also located in areas with higher canopy cover than those without any foraging trail (with foraging trail/s: 61.83% ±3.73, without foraging trail: 56.71% ±2.62, Mean ± SE, linear mixed-effect model, fixed effect: t = 4.57, *P*<0.001, n = 201, model AIC = 1443.27). The minimum length of foraging trails decreased with an increase of canopy cover (minimum length of foraging trails: 4.52 m±0.33, Mean ± SE, linear mixed-effect model, fixed effect: t = –4.44, *P*<0.001, n = 135, model AIC = 979.40). There was no relationship between minimum foraging trail length and nest size (linear mixed-effect model, fixed effect: t = –0.66, *P*<0.51, n = 135, model AIC = 309.46).

**Figure 5:**
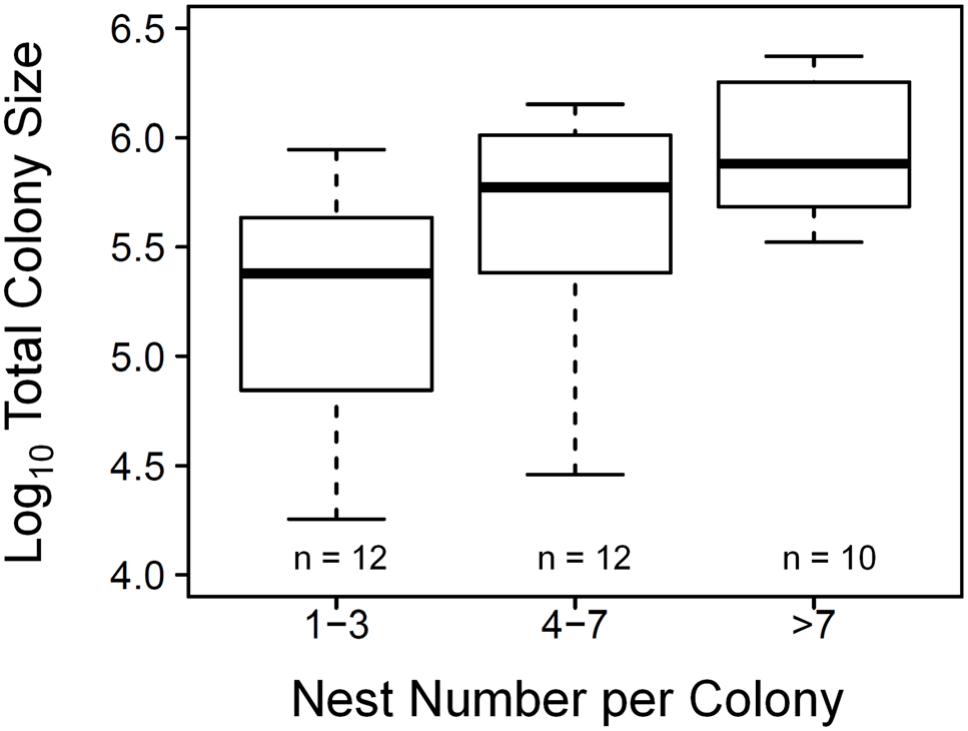
The relationship between number of nests and total colony size. Kruskal-Wallis’ test, *χ* ^2^ = 10.15, *P*<0.01.

**Figure 6:**
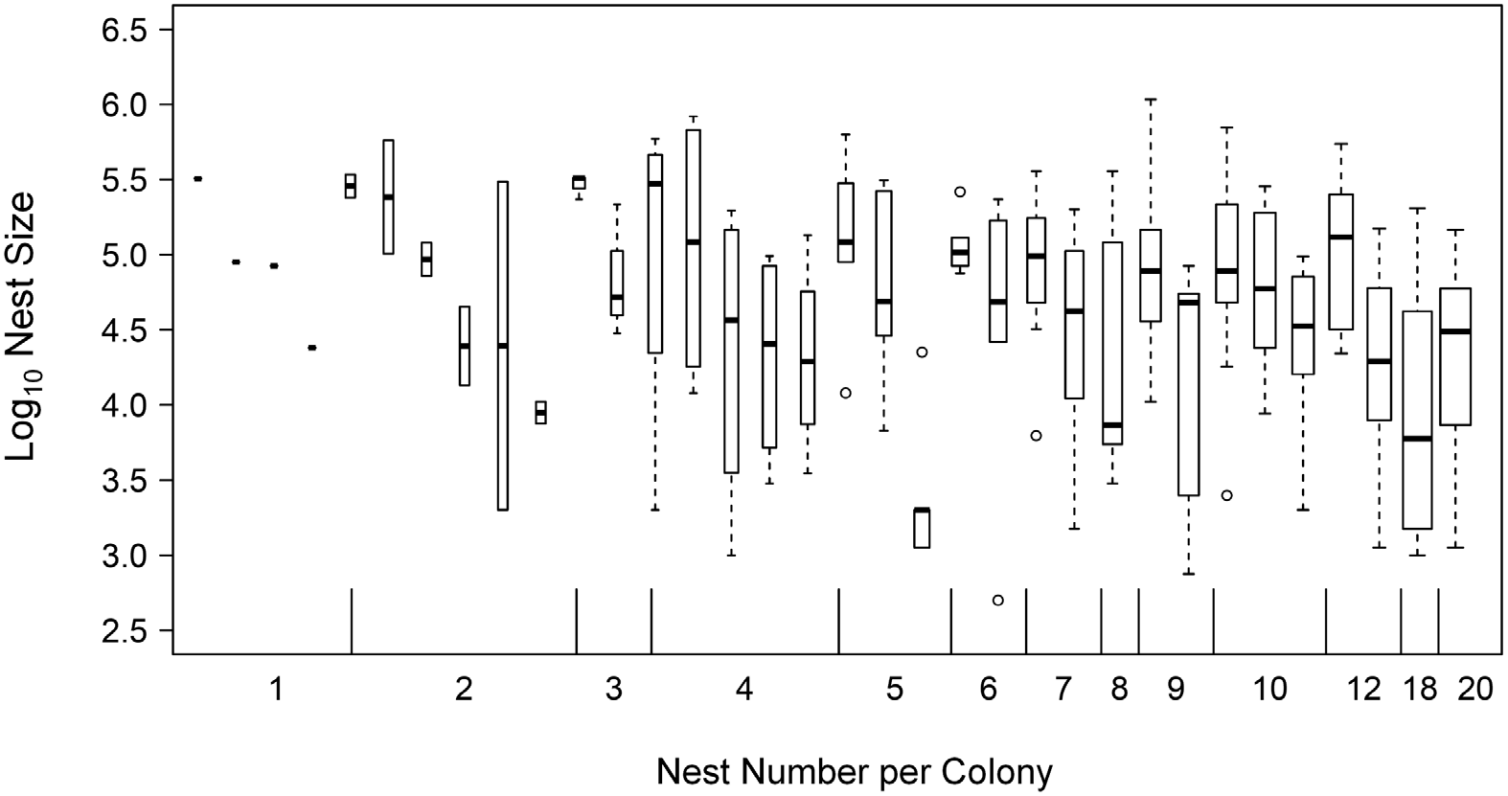
The relationship between number of nests per colony and nest size. Nest size tends to decrease as number of nests per colony increases (Linear mixed effect model, fixed effect: t = –2.03, *P* = 0.051, model AIC = 462.33). Boxplot width is proportional to the square root of number of nests.

**Figure 7:**
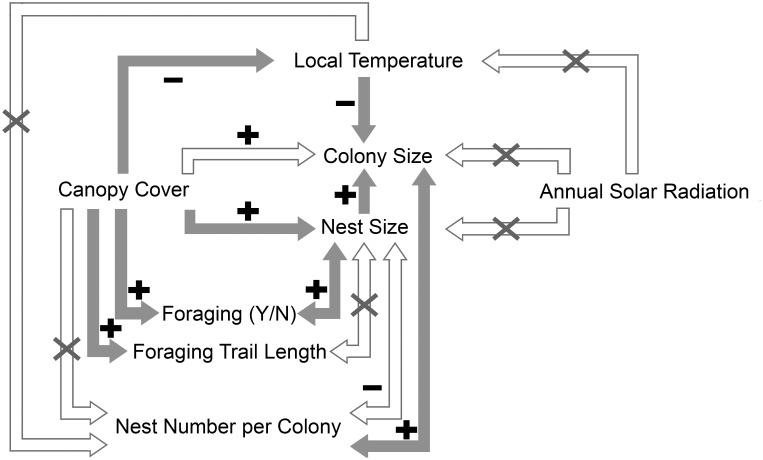
The relationships of colony size and nest size to possible related factors in our study. Arrows illustrate the possible direction of causality. Solid arrow, hollow arrow and hollow arrow with a cross indicate the significant, the borderline significant and the non-significant relationships respectively. Plus and minus signs indicate the relationships as positive and negative respectively.

Colonies included in this study ranged from a single nest (monodomous) to as many as 20 nests connected as a single polydomous colony. Total colony size of a polydomous colony could be larger through one or both of the following ways: have bigger individual nests, or have more nests per colony. Our results showed that colonies with greater total colony size had greater mean nest size (mean nest size value as Fig. 1 shows, *F* = 40.41, d.f. = 33, *P*<0.001, *r* ^2^ = 0.56). On the other hand, total colony size also increased when a colony had more nests; there was a significant increase in total colony size from Close-to-Monodomous Group (with one to three nests, n = 12) to Intermediate-Polydomous Group (with four to seven nests, n = 12) and Polydomous Group (with more than seven nests, n = 10) (Kruskal-Wallis’ test, *χ^2^* = 10.15, *P*<0.01, Fig. 5). Three groups did not significantly differ in the variances of total colony size from each other (Levene’s test, *F* = 1.88, *P* = 0.16). As for the two factors which are related to total colony size, we found a borderline significant negative relationship between nest size and the number of nests per colony (linear mix-effect model, fixed effect: t = –2.03, *P* = 0.051, n = 201, model AIC = 462.33, Fig. 6). There was no significant relationship between canopy cover and number of nests per colony (linear mix-effect model, fixed effect: t = –1.00, *P* = 0.33, n = 201, model AIC = 1463.63). Number of nests per colony had no significant relationship with the six local temperature parameters (linear regressions, *F* = 0.00–0.38, d.f. = 32, *P* = 0.54–0.95). Annual solar radiation had no significant relationship with the six local temperature parameters (linear regressions, *F* = 0.02–0.95, d.f. = 32, *P* = 0.34–0.88). We made a flow chart showing the relationship between canopy cover, nest size, colony size and other factors in the present study (Fig. 7).

## Discussion

Our results clearly showed that red wood ant (*F. lugubris*) nest size increased with increasing canopy cover: nests in shady areas were larger than those in open areas (Fig. 1). Temperature and food resources are two important factors which would be predicted to affect nest size and are related to canopy cover. Impacts of the thermal environment on nest size could be mediated through two routes: thermoregulation and worker population dynamics. For thermoregulation, a wood ant nest with a small worker population has to rely on direct sun radiation to reach and maintain a sufficient inner nest temperature [67], [68]. In contrast, thermoregulation of nests with larger worker populations can be independent of sun exposure because of sufficient endogenous heat generation, based on the metabolism and clustering behaviour of workers [58], [69] and microbial heat production within the nest material [69], [70]. In terms of worker population dynamics, brood development rate and the egg production rate of queens increase with increasing temperature; meanwhile, worker longevity decreases [18], [19]. The trade-off between brood developmental rate, egg reproductive rate and worker longevity determines how the nest grows in size, which is related to the potential of producing sexual offspring [71].

Our temperature measurements showed that the thermal environment of areas with higher canopy cover was generally colder and more stable than that of more exposed areas (Fig. 3). To cope with the cold, nests in shady areas must be large enough to execute effective thermoregulation. Among our sampling points, the maximum canopy cover was 86%. This means that even in the shadier areas nests may sometimes receive sunshine. When sunshine falls on the nest, it might not cover the whole nest mound. This could cause a thermal gradient in the stable cool environment of shadier areas. Therefore, when the sunshine is present, the shadier areas provide a nest with greater variety of thermal environments aiding regulation of worker population dynamics: workers could stay in cooler chambers for longer longevity and could move brood to warmer part for a faster development rate. Shady areas not only necessitate nest growth but could also actively promote it.

For the nests in open areas, although the mean daily minimum temperature is a little lower, the mean daily maximum is much higher than that in shadier areas (Fig. 3). The whole or a large part of the nest passively experiences a generally warmer environment, so the nest has less need to grow larger to allow thermoregulation. In addition, warmth-related increases in egg production and brood developmental rate might not be able to compensate for the decreased worker longevity. Thus the nests in very open areas were smaller than those in shady areas. There may also be additional influences from the growth-stage-related thermal requirements for wood ant nests and the forest succession: a newly-built or young wood ant nest is usually small therefore might not survive in shadier areas, and the dynamics of forest succession can result in the canopy modification. Overall, local temperature was probably the primary mediating factor for the relationship we found between canopy cover and nest size. Modelling nest size growth in different thermal environments could be a feasible approach for future studies of red wood ants, with physiological data related to temperature, for example, the relationship of temperature with worker longevity, brood developmental rate and queen’s egg production.

In addition to temperature, food resource availability is another factor which influences wood ant nest size [54], [55]. The majority of the ants in the trails connecting trees and nests are foragers, which collect honeydew from aphids (more than 90% of a colony’s nutrition) [29], [72]. Low canopy cover may therefore imply a decrease of available foraging trees for wood ant nests. Our study showed that nests with foraging trails were generally located in shadier areas and nests without foraging trails in more exposed areas. Among the nests with foraging trail/s, minimum foraging trail length was shorter in shady areas than that in open areas, which, as would be expected, indicated that nests were closer to trees in shady areas than in open areas. Nests with foraging trail/s also were larger than nests without foraging trail/s (Fig. 4). This matches the findings of a previous study at the same site using a partially overlapping sample set, which also found that *F. lugubris* nests with foraging trail/s were larger and in shaded areas than nests without any foraging trail [62].

Although we might be able to assume that shadier areas provided more possible food resources resulting in the presence of foraging trails, the direction of causality between nest size and the presence of foraging trails is not clear (Fig. 7). On one hand, an established foraging trail may provide more food to promote nest growth. On the other hand, an alternative hypothesis is that only nests above a certain size are able to establish and maintain a lasting foraging trail. Our data showed that although nests with foraging trail/s were on average bigger than those without a trail, the minimum nest size was similar for nests both with and without foraging trails (Fig. 4). This would seem to rule out the existence of a nest size threshold which determines whether a nest starts foraging or not, at least within our observed range of nest sizes, and so it is quite possible that the presence of one or more foraging trails promotes increased nest size. Therefore, in addition to local temperature, food resource availability is another possible mediating factor for the relationship between canopy cover and nest size. Interestingly, we only found a few small nests in highly shady areas (for example, over 70% canopy cover, see Fig. 1). Food resources are unlikely to be limiting in these areas, so there should be other reasons why small nests are less common. For example, if a new nest in a highly shady area does not grow over a “threshold” size, it may not survive over the winter. It seems that the thermal effects of canopy cover are more important than the relationship with food resource availability, in terms of nest size. We therefore suggest an initial mechanistic process when a nest is newly built: higher canopy cover implies nearer trees resulting in higher food resource availability, and the effects of the thermal environment on worker population dynamics promotes nest growth. The benefits of larger nest size for thermoregulation could result in a positive feedback on nest growth once the nest reaches a certain size. Further work is needed to investigate the relative importance of these different effects over the course of colony establishment, growth and maturity.

At the colony level, we found a trend that total colony size increased with increasing canopy cover. Total colony size was also related to local temperature in the same way. The trend between total colony size and canopy cover was eliminated when a three-way partial correlation was applied to remove the effects of local temperature. These results indicate that, similar to the nest level, local temperature seems to be a mediating factor between canopy cover and total colony size (Fig. 7). Furthermore, annual solar radiation had no effect on total colony size nor local temperature in our study; this further supports that the thermal environment experienced by wood ant colonies was strongly determined by canopy cover in this flat area. If higher canopy cover results in increasing total colony size, this could occur in two ways: a polydomous colony has larger total colony size either because it has bigger nests, or because it has more nests, or both. For the first way, we found that a colony that had larger total colony size also had larger mean nest size. This suggests that canopy cover probably influences total colony size through the thermal effects on nest size discussed above. Apart from nest size, our results also showed that total colony size increased when the number of nests increased (Fig. 5). For these reasons, we suggest that a polydomous wood ant colony may increase total colony size by both ways: increasing the size of each nest and increasing the number of nests, but mainly by the former. We also suggest that these two approaches compensate for the effects from each other because a there was a borderline significant negative trend between the nest size and the number of nest per colony (Fig. 6). Moreover, neither canopy cover nor local temperature was related to number of nests per colony (Fig. 7). It seems that if the canopy cover has impacts on the qualities of the environment (eg: local temperature or food resource abundance) that affect total colony size, it acts more at the nest level (individual nest size) than at the colony level (the number of nests per colony).

This paper presents a study specifically focused on the relationships of canopy cover to ant nest size and colony size. Our results support and strengthen a marginally significant trend between canopy cover and nest size which was found at the same site by Ellis et al. [62]. The stronger finding in our study is probably due to methodological differences. First, Ellis et al. [62] actively chose the largest ten colonies for a nest network study; in our study an even-distribution survey was performed. Second, Ellis et al. used images from digital photographs for canopy cover; in our study the circle images of sky which were taken by a fisheye lens provided a complete estimation of canopy cover. Third, colony identity was included in our analyses. Frouz and Finér [67] also found similar relationships between nest size and canopy cover in another red wood ant species *Formica polyctena*. This study again focussed only on the nest level, and used a semi quantitative scale to estimate shading, which differentiates three levels of shading by daily sunshine hour [69].

In regard to the canopy-related relationships between polydomy and colony-level organisation, previous studies have showed two different results. Sorvari and Hakkarainen [55] reported a higher degree of polydomy in *F. aquilonia* in clear-cut areas where the colonies experienced an extreme environment. They hypothesised that new nests are established by budding more frequently in clear-cuts than in forest interior in order to be near the forest edge for food resources. In contrast, Punttila [73] suggested that monogynous (monodomous) populations of *F. lugubris* should be common in young forest before the canopy closure, whereas polygynous (polydomous) *F. aquilonia* should dominate in older forests and in the interior areas. He suggested a mechanism from inter-specific competition and forest succession: with bigger size of the dispersing females, *F. lugubris* is a more efficient coloniser than is *F. aquilonia*. Female *F. lugubris* disperse to a young forest first where the canopy is still open, and *F. aquilonia* dominates over other species when it comes in the gradually mature forest later by nest budding. Another survey for several mound-building species (including *F. lugubris* and four red wood ant species) was conducted by Punttila and Kilpeläinen [54] in Finland. They found species-specific associations of nest size with canopy cover. In our study, neither a positive nor negative relationship between canopy cover and the number of nests was found. We suggest that the impacts from canopy cover acts on the nest level rather than on the colony level. This further supports the finding of Ellis et al. [62] which also found no relationship between the number of nests and canopy cover (10 colonies with a total of 140 nests). Overall, the differences between studies may result from the differences between sampling sites and between methodologies, for example, whether other wood ant species are present or not, whether the ants are experiencing normal forest succession or extreme events such as clear-cutting, and whether the studies are focused within or across species.

The most direct approach of understanding the influence of an environmental factor on a species is probably to examine their physiological or life-history characteristics in direct response to the environmental factor, for example, temperature. However, a laboratory approach has some limitations for our question. Investigating the relationship between canopy cover and wood ant nest size in the field solves it in many aspects. First, it is not feasible to simulate the daily or annual temperature in a laboratory approach because the exact dynamics are complex. Canopy cover provides an index for estimating local thermal environment. Second, we can obtain the nest size data in a natural environment with little disturbance to colony function and organisation. Moreover, the present study shows an overall reaction of wood ant nests to canopy cover. Canopy cover may influence nests by changing not only the features of temperature but also the food resource availability (Fig. 7). Future studies could involve canopy manipulation or the seasonal variation in canopy cover to monitor the long-term change in nest size and the colony-level organisation on wood ant species, which are ecologically significant in the forest ecosystem. As the effect of climate change on species can act through multiple and complex ways (changes in vegetation, species interaction and human activity), species-specific responses to future climate change are challenging to predict. A prediction based on causal relationships between the environments (eg: canopy cover) and the life-history characteristics (eg: nest size and colony size) may suggest possible future outcomes, thus help species’ conservation and potentially reduce negative impacts of climate change on these species.
